# The epileptogenic zone in children with tuberous sclerosis complex is characterized by prominent features of focal cortical dysplasia

**DOI:** 10.1002/epi4.12529

**Published:** 2021-08-06

**Authors:** Hanna M. Hulshof, Barbora Benova, Pavel Krsek, Martin Kyncl, Maarten H. Lequin, Anezka Belohlavkova, Petr Jezdik, Kees P. J. Braun, Floor E. Jansen

**Affiliations:** ^1^ Department of Pediatric Neurology Brain Center University Medical Center Utrecht Utrecht The Netherlands; ^2^ Department of Pediatric Neurology Second Faculty of Medicine Charles University and Motol University Hospital Prague Czech Republic; ^3^ Department of Radiology Second Faculty of Medicine Charles University and Motol University Hospital Prague Czech Republic; ^4^ Department of Radiology Brain Center University Medical Center Utrecht Utrecht The Netherlands; ^5^ Department of Circuit Theory Faculty of Electrical Engineering Czech Technical University Prague Czech Republic

**Keywords:** epilepsy surgery, focal cortical dysplasia, magnetic resonance imaging, pre‐surgical evaluation, tuberous sclerosis complex

## Abstract

**Objective:**

Patients with tuberous sclerosis complex (TSC) present with drug‐resistant epilepsy in about 60% of cases, and evaluation for epilepsy surgery may be warranted. Correct delineation of the epileptogenic zone (EZ) among multiple dysplastic lesions on MRI represents a challenging step in pre‐surgical evaluation.

**Methods:**

Two experienced neuroradiologists evaluated pre‐ and post‐surgical MRIs of 28 epilepsy surgery patients with TSC, assessing characteristics of tubers, cysts, calcifications, and focal cortical dysplasia (FCD)—resembling lesions. Utilizing multiple metrics, we compared MRI features of the EZ—defined as the resected area in TSC patients who achieved seizure‐freedom 2 years after epilepsy surgery—with features of other brain areas. Using combinatorial analysis, we identified combinations of dysplastic features that are most frequently observed in the epileptogenic zone in TSC patients.

**Results:**

All TSC‐associated dysplastic features were more frequently observed in the EZ than in other brain areas (increased cortical thickness, gray‐white matter blurring, transmantle sign, calcifications, and tubers; Kendal's tau 0.35, 0.25, 0.27, 0.26, and 0.23, respectively; *P* value <.001 in all). No single feature could reliably and independently indicate the EZ in all patients. Conversely, the EZ was indicated by the presence of the combination of three of the following features: tubers, transmantle sign, increased cortical thickness, calcifications, and the largest FCD‐affected area. Out of these, the largest FCD‐affected area emerged as the most reliable indicator of the EZ, combined either with calcifications or tubers.

**Significance:**

The epileptogenic zone in TSC patients harbors multiple dysplastic features, consistent with focal cortical dysplasia. A specific combination of these features can indicate the EZ and aid in pre‐surgical MRI evaluation in epilepsy surgery candidates with TSC.

**Key points**

1. The epileptogenic zone (EZ) of tuberous sclerosis complex (TSC) patients shows MRI features of focal cortical dysplasia (FCD)
2. No single focal cortical dysplasia‐like feature can reliably identify the EZ on MRI of TSC patients but the combination thereof can
3. Increased cortical thickness, transmantle sign, blurring of the gray/white matter, tubers and calcifications occur most often in the EZ

## 1 INTRODUCTION

Tuberous sclerosis complex (TSC) is an autosomal‐dominant multisystem disorder, caused by mutations in the *TSC1* or *TSC2* gene. Epilepsy occurs in 90% of all TSC patients, with the onset before the first year of life in 62.5%‐73% and in 78% before the second.^1^, ^2^, ^3^ Early and complete seizure control is significantly related to improved development in TSC patients.^4^ In almost two thirds of patients, seizures do not respond to medical treatment, and epilepsy surgery needs to be considered. Previous studies reported seizure freedom in up to 55%‐60% of TSC patients after epilepsy surgery.^5^, ^6^, ^7^

Localization of the epileptogenic zone (EZ) during pre‐surgical evaluation is challenging in TSC patients, due to the presence of multiple dysplastic lesions and often multiple seizure types and multifocal EEG abnormalities. Several non‐invasive tests are undertaken in the preoperative evaluation of TSC patients. Patients with discordant or uncertain findings from non‐invasive evaluation are either refused epilepsy surgery or proceed to invasive EEG monitoring to localize and delineate the EZ.^8^ Despite advances in electrophysiological and functional neuroimaging studies, structural MRI remains the cornerstone of pre‐surgical evaluation in epilepsy patients that guides further decision‐making.

Few studies have proposed potential neuroimaging markers of epileptogenicity in TSC, including tuber size,^9^ calcifications,^10^ cystic changes,^11^ and an increased apparent diffusion coefficient (ADC) of epileptogenic compared to non‐epileptogenic tubers.^12^, ^13^ In their study, Jahodova et al^14^ showed that an experienced neuroradiologist was able to identify resection area on pre‐surgical brain MRI in all seizure‐free patients, based on the local accumulation of features consistent with focal cortical dysplasia (FCD). However, they did not identify specific combinations that would most reliably associate with the EZ among the myriad of dysplastic lesions observed in a brain MRI of a single patient.

In our study, we therefore aimed to identify features of the EZ in TSC patients that could—either isolated or in combination—distinguish the EZ from other cortical areas with TSC‐associated changes outside the EZ and therefore aid in the pre‐surgical evaluation of TSC patients.

## 2 METHODS

### 2.1 Study cohort

We included patients with a definite diagnosis of TSC,^15^ aged 12 months or older, who underwent resective or disconnective—intentionally curative—epilepsy surgery in the University Medical Center Utrecht (UMCU) or in Motol University Hospital Prague (MUH), between 2003 to 2015. Patients who underwent intended palliative surgery (eg, corpus callosotomy and implantation of vagal nerve stimulator) were excluded. Only patients who had (a) at least one preoperative and one postoperative MRI of adequate technical quality (see below for technical specifications); and (b) known postoperative seizure outcome with a follow‐up duration of at least 2 years were included.

All patients underwent pre‐surgical examinations as considered necessary by the multidisciplinary epilepsy surgery team. The epileptogenic zone to be resected was identified by the team as a result of a complex diagnostic process, including electrophysiology, anatomical and functional neuroimaging, and neuropsychology. Postoperative seizure outcome was classified using Engel‐classification scale (I‐IV) at the end of follow‐up period of minimum 2 years.

The study was approved by the medical ethics committee of the UMCU, who judged that the Medical Research Involving Humans Act (WMO) did not apply, and approved by the Motol University Hospital ethics committee.

### 2.2 MRI analysis

At the start, two radiologists experienced in pediatric neuroradiology (MK, MHL) agreed to a set of predefined technical specifications of MRI protocols to render them appropriate for MRI analysis, including MRI field strength and given sequences. Based on these requirements, we included for further analyses MRIs of 1.5T or 3T field strength with at least the following sequences: T2 FLAIR in coronal, and axial or sagittal directions, max 5 mm slice thickness (in most cases <5 mm), T1 weighted sequences in sagittal and axial directions and a T2 weighted axial sequence, max 5 mm slice thickness (in many cases 3D scans). For the sake of review and analysis, the brain was divided into 11 predefined cortical regions of interest (ROIs) per hemisphere: frontal (mesial, lateral, polar, basal, and central), temporal (mesial and lateral), parietal (mesial and lateral), and occipital (mesial and lateral).

Using a training set of MRIs from three patients with TSC who were not included in the cohort studied here, the radiologists agreed on what constituted specific TSC‐associated dysplastic features to be evaluated, based on established definitions (see below). Then, both radiologists independently evaluated all MRIs from both centers. They were blinded to all clinical and outcome data, including site of resection, when assessing preoperative MRIs.

**FIGURE 1:**
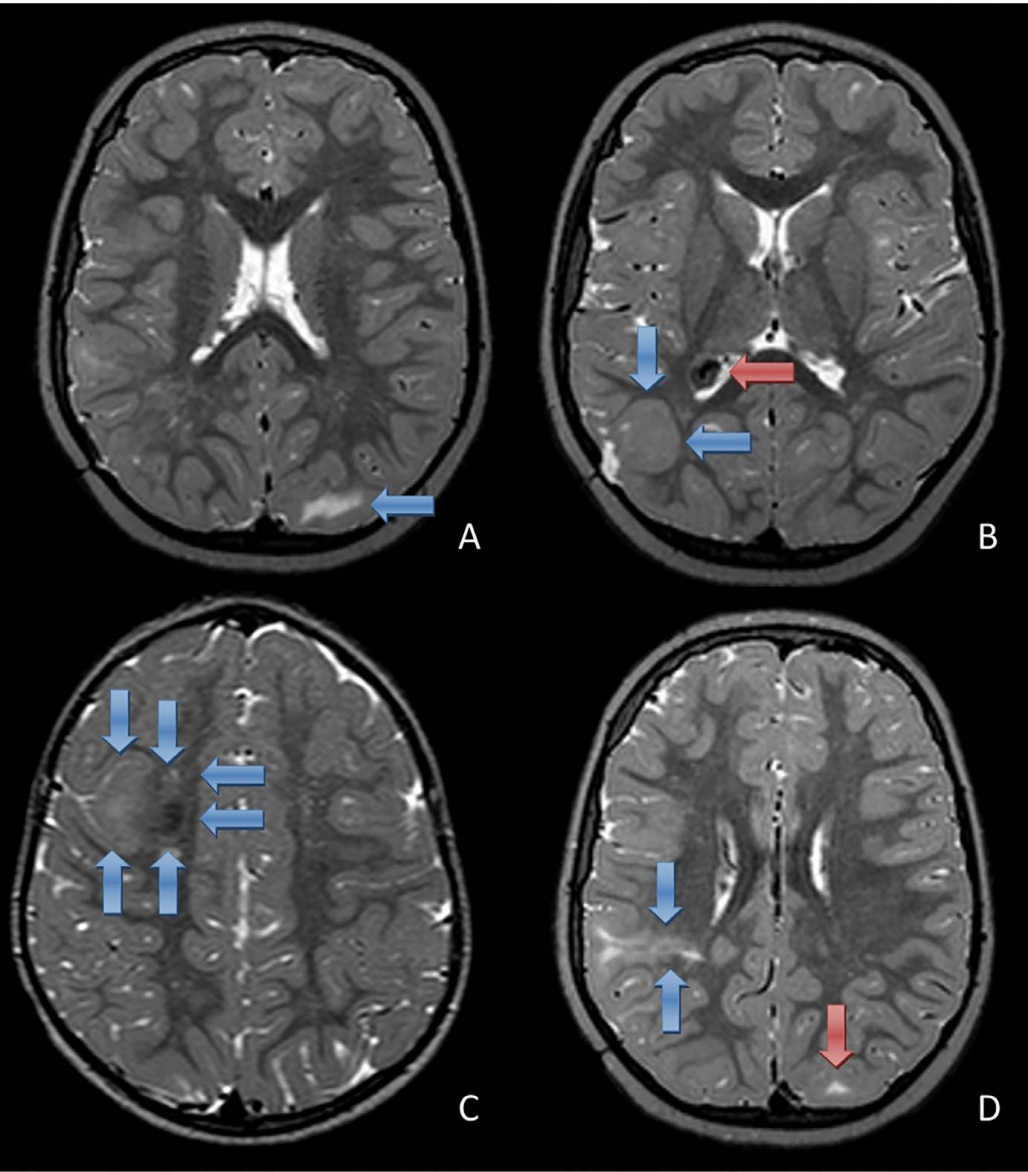
MRI abnormalities in TSC, shown on T2 weighted images. Tuber in the left occipital lobe, seen as a hyperintense lesion (blue arrow, A). Increased cortical thickness in the right parieto‐occipital region (blue arrows, B). Also, a SEGA is shown in the lateral ventricle, unrelated to patient's epilepsy (red arrows, B). Blurring of the gray‐white matter junction with a calcification in the right frontal lobe (blue arrows, C). Transmantle sign in the right parietal lobe, seen as a subcortical hyperintense lesion with a tail extending toward the ventricle (blue arrows, D). Also, a tuber is shown in the left occipital lobe, seen as a small subcortical hyperintense lesion (red arrow, D)

Each cortical ROI was assessed for the presence or absence of the following abnormalities: (a) tubers, defined as areas of increased signal intensity in subcortical or cortical regions on T2‐weighted or T2 FLAIR images^16^; (b) cystic lesions, defined as hypointense on T1, hyperintense on T2 and hypointense or heterogeneous on FLAIR, on which they were characterized by a hypointense central region surrounded by a hyperintense rim^17^, ^18^; and (c) calcifications, defined as hypointense on T2, in most cases hyperintense on T1 and heterogeneous on FLAIR characterized by a hypointense central region surrounded by a hyperintense rim (Figure 1).^18^ It is well known that tubers can express FCD‐like features themselves, but in addition to tubers other dysplastic lesions can be found in TSC patients. Therefore, all brain ROIs, not only the regions surrounding tubers, were evaluated for the presence or absence of the following FCD‐like features: increased cortical thickness, blurring of the gray‐white matter (GWM) junction and a transmantle sign, defined as an area of abnormal signal corresponding to the signal of gray matter extending from the cortex to the superolateral wall of the lateral ventricle.^19^ Furthermore, the ROI containing the largest FCD‐affected area was denoted. Figure 1 shows examples of MRI scans with the respective features.

### 2.3 Study design

Both neuroradiologists were first instructed to localize and score the above‐defined MRI abnormalities in the 22 predefined brain areas on pre‐surgical MRI scans. The epileptogenic zone was operationally defined as the resected area in patients who were and remained seizure‐free after at least 2 years of follow‐up. In these seizure‐free patients, we compared the features of the (resected) epileptogenic zone with all other brain ROIs and analyzed (a) whether any of the aforementioned features was significantly associated with the EZ; and (b) whether there was a particular combination of features that characterized the EZ.

### 2.4 Statistical analysis

Demographical data were presented using descriptive statistics. *P* value < 0.05 was considered statistically significant.

First, we calculated Cohen's kappa for all observed features to assess the agreement between the two radiologists. Second, we used Kendal's tau as a correlation metrics to show whether there was a statistically significant relationship between a feature's presence in any ROI vs the EZ. In addition, we calculated metrics that can show the reliability of each feature in terms of its probability of correct and false discoveries (accuracy, positive predictive value, and false discovery rate). These calculations were performed for all the ROIs of the seizure‐free patients among the datasets generated by each neuroradiologist and were compared between each other. In the next step, we calculated the same metrics separately for each patient and each radiologist's assessment. After analyzing each feature separately, we utilized a combinatorial analysis to assess the predictive value of various combinations of the analyzed features to correctly identify the EZ for each radiologist. The combinations were tested as an optimization task by brute force algorithm with the EZ as a reference (link to the script can be found on: https://github.com/jezdip1/tsc_mri_predictors_of_ez). For the calculations, software MATLAB version 2017b and its statistical computing toolbox was used.

## 3 RESULTS

### 3.1 Clinical cohort

A total of 28 TSC patients (10 from MUH and 18 from UMCU) who underwent resective epilepsy surgery fulfilled the inclusion criteria and their MRIs were evaluated. A tailored focal resection was performed in 18 patients, five underwent lobar resections, four had a multilobar resection and one a hemispherotomy. Mean age at the onset of clinical seizures was 12.3 months (range 0.07‐79 months), 82% of patients had daily seizures during pre‐surgical evaluation. Median age at surgery was 9.3 years (range 1‐47 years). In 10 patients, prior invasive monitoring was performed, followed by resective surgery. In the total cohort, 24 operations were guided by ECoG. Mean duration of follow‐up was 5.2 years (range 2.0‐14.0 years). Fifteen patients (53.6%) were seizure‐free at last follow‐up. AEDs were successfully withdrawn in eight patients. Seizure outcome in the remaining patients was classified as follows: seven patients (25%) Engel II, three patients (10.7%) Engel III, and three patients (10.7%) Engel IV.

### 3.2 MRI findings in all patients

All 28 patients exhibited numerous MRI abnormalities. Tubers were present in all patients, cysts, and calcifications in only 21.4% and 39.3% of patients, respectively. In all patients, at least one of the FCD‐like features was present, in most cases, limited to a certain number of brain areas. In total, 616 ROIs were reviewed, but only 244 contained abnormalities: 52.5% of ROIs contained one abnormality, 30.3% two, 11.1% three, 4.1% four, and 2% five abnormalities. An overview of MRI findings is presented in Table 1.

**TABLE 1:** Summary of MRI findings in the whole cohort

MRI findings	All patients *n* = 28 (%)	All ROIs *n* = 616 (%)
Tubers	28 (100%)	187 (30.3%)
Cysts	6 (21.4%)	11 (1.8%)
Calcifications	11 (39.3%)	13 (2.1%)
Increased cortical thickness	21 (75.0%)	34 (5.7%)
GWM junction blurring	26 (92.9%)	110 (17.9%)
Transmantle sign	24 (85.7%)	65 (10.6%)

Abbreviation: GWM, gray‐white matter.

### 3.3 Agreement between the two radiologists

Except for the assessment of cysts, the two neuroradiologists achieved fair or substantial agreement in all tested features (GWM blurring, Transmantle sign, Increased thickness, Calcifications, Largest FCD‐affected area, Tuber; *P* < .001).

### 3.4 MRI features of the EZ in seizure‐free patients

For the analysis of the EZ, we only included patients who were seizure‐free at the 2‐year follow‐up (n = 15). Each patient was assessed for 22 regions; therefore, the total number of regions assessed was 330 (22 × 15). Table 2 shows the association between respective features and the EZ (for all ROIs) as assessed by each radiologist and the correlation between the two neuroradiologists. The table denotes the value of Kendal's Tau for each feature as a measure of correlation between the actual EZ and the area considered EZ by the respective radiologist. For example, the presence of a transmantle sign—as evaluated by MHL—correlated significantly more with the EZ than with the other ROIs and therefore differentiated that particular region as the EZ from all other ROIs (KT 0.0.27, *P* < .001). The last column denotes the level of correlation between the two radiologists.

**TABLE 2:** Summary of results of correlation between respective features and the EZ

Metrics features	MHL's evaluation of EZ		MK's evaluation of EZ		MK vs MHL	
	KT	*P* value	KT	*P* value	KT	*P* value
No of abnormalities	0.27	<.001	0.31	.010	0.69	<.001
No. of abnormalities (incl. largest affected area)	0.27	<.001	0.302	.011	0.697	<.001
GWM blurring	0.25	<.001	0.25	<.001	0.33	<.001
Transmantle sign	0.27	<.001	0.23	<.001	0.36	<.001
Increased thickness	0.35	<.001	0.29	<.001	0.60	<.001
Cysts	−0.03	.544	−0.02	.672	−0.01	.891
Calcification	0.26	<.001	0.65	<.001	1.00	<.001
Largest FCD‐affected area	0.40	<.001	0.39	<.001	0.90	<.001
Tuber	0.23	<.001	0.14	.013	0.60	<.001
No. of abnormalities in ROI (excl. calcifications)	0.27	<.001	0.30	.011	0.70	<.001

Abbreviations: EZ, epileptogenic zone; GWM, gray‐white matter; KT, Kendal's tau; MHL, Maarten H. Lequin; MK, Martin Kyncl.

Next, we assessed the accuracy, positive predictive value (PPV), and false discovery rate (FDR) for each feature as assessed by each of the neuroradiologists for all 330 regions as summarized in Table 3.

**TABLE 3:** Summary of the accuracy, positive predictive value, and false discovery rate for each feature

Metrics features	MHL's evaluation of EZ			MK's evaluation of EZ		
	Accuracy	PPV	FDR	Accuracy	PPV	FDR
GWM blurring	84%	25%	75%	87%	29%	71%
Transmantle sign	89%	35%	65%	88%	30%	70%
Increased thickness	92%	53%	47%	89%	34%	66%
Cysts	90%	0%	100%	91%	0%	100%
Calcification	92%	75%	25%	97%	67%	33%
Largest FCD‐affected area	92%	60%	40%	92%	56%	44%
Tuber	62%	16%	84%	52%	12%	88%

Abbreviations: EZ, epileptogenic zone; FDR, false discovery rate; GWM, gray‐white matter; MHL, Maarten H. Lequin; MK, Martin Kyncl; PPV, positive predictive value.

To summarize, all FCD‐like features, tubers, calcifications, and the ROI with the largest FCD‐affected area and the ROI with the most abnormalities present were significantly associated with the EZ. The features “calcifications” and the “largest FCD‐affected area” showed the greatest accuracy in predicting whether an ROI is the EZ.

### 3.5 Association between analyzed features and the EZ calculated for patients individually

**FIGURE 2:**
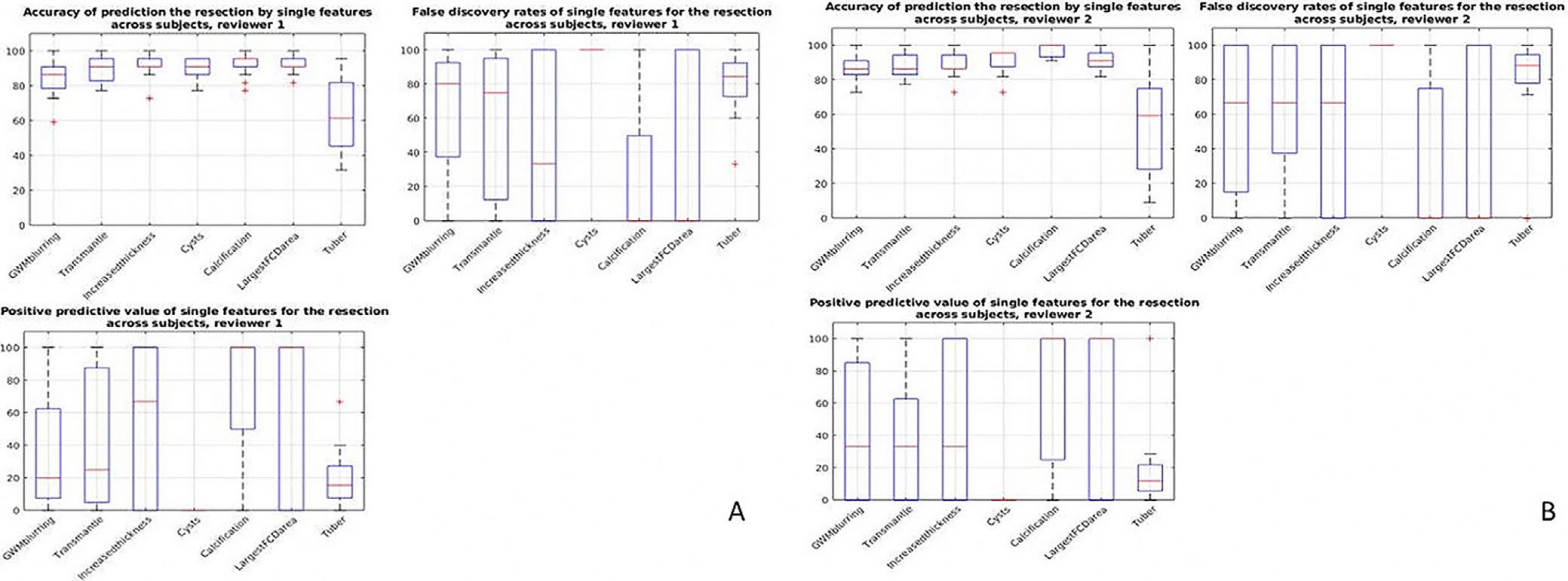
Positive predictive value (PPV), false discovery rate (FDR), and accuracy across subjects for neuroradiologist 1 (A, MHL) and 2 (B, MK)

In the next step, we calculated the PPV, FDR, and accuracy for each of the features—the values show the accuracy and PPV for each feature to correctly identify the EZ. FDR of each feature represents a measure of chance for incorrectly identifying the EZ. The values were calculated separately for each particular patient; this decreased the number of regions assessed in every calculation to 22. Results summarized for all subjects are plotted onto the graphs in Figure 2. In summary, there is no single predictor capable of reliably identifying the EZ across all subjects in the study.

### 3.6 Which combination of analyzed features can predict the EZ most accurately?

Based on a brute force combinatorial algorithm, it was calculated that the visual scoring of neuroradiologist 1 could identify at least one ROI correctly as the EZ with 100% (PPV) in 10 of 15 patients under the following conditions: presence of at least three of the five following MRI features (transmantle sign, increased cortical thickness, calcifications, largest FCD‐affected area, tuber); presence of at least two of the following four MRI features (transmantle sign, increased cortical thickness, calcifications, largest FCD‐affected area) or presence of at least one of the two following features (calcifications, largest FCD‐affected area).

**FIGURE 3:**
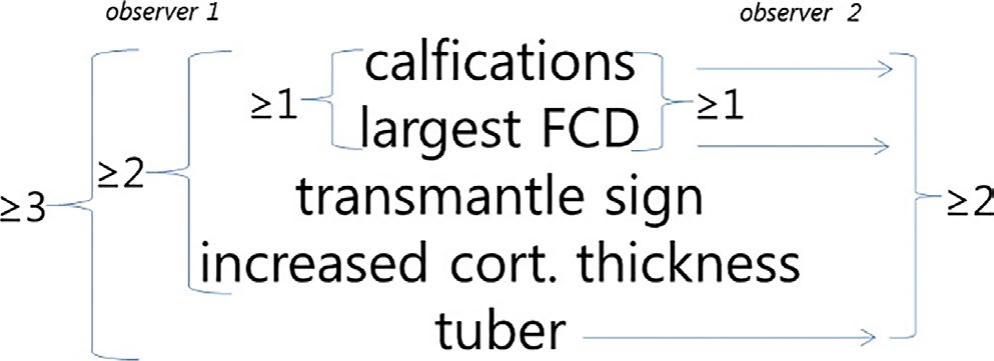
Combinations of various features that can correctly identify the epileptogenic zone for each of the neuroradiologists

Based on the same algorithm, it was calculated that the visual scoring of neuroradiologist 2 could identify at least one ROI correctly as the EZ with 100% (PPV) in 10 of 15 patients under the following conditions: presence of at least two out of three features (calcifications, largest FCD‐affected area, tuber) and presence of at least one of the two features (calcifications, largest FCD‐affected area). Results are summarized in Figure 3.

## 4 DISCUSSION

In this study, we analyzed whether any of the radiological features of TSC—in particular those consistent with focal cortical dysplasia—either isolated or in combination, could identify the epileptogenic zone in TSC patients undergoing pre‐surgical evaluation. Based on our findings, we identified multiple combinations of dysplastic features that characterized the epileptogenic zone on the pre‐surgical MRI of TSC patients.

### 4.1 Characteristic features of the EZ in TSC patients

In comparison with all other affected brain regions, the epileptogenic zone (operationally defined as the resected area in patients who were and remained seizure‐free after 2 years of follow‐up) harbored a larger number of features typical of FCD (increased cortical thickness, gray/white matter blurring, transmantle sign), tubers, and calcifications. In the majority of cases, the EZ also represented the largest area affected with dysplastic changes. Although no single feature was shown to reliably mark the EZ, various combinations of MRI features predicted the localization of the EZ with sufficient accuracy. We also observed that certain features, for example, calcifications and largest FCD‐affected area, carry more weight in the assessment process, as the top of the decision‐making tree.

Our findings confirm and extend those from Jahodova et al,^14^ who correctly identified the epileptogenic zone on structural MRI alone in all seizure‐free patients and found that it was characterized by multiple FCD‐like features. In addition, our findings are consistent with those previously reported, showing that the EZ in TSC patients more often contains calcifications compared to non‐EZ brain areas.^10^ Unlike previous studies,^11^ we did not find an association between the presumed epileptogenic zone and the presence of cystic lesions.

### 4.2 No single feature can reliably predict the localization of the EZ

Most previous studies have focused on identification of a single marker of epileptogenicity among the myriad of dysplastic changes observed on brain MRIs of TSC patients that render the MRI evaluation so challenging. However, our study shows that there is no single abnormality that could unequivocally be considered “epileptogenic” and could predict post‐surgical seizure freedom, after complete resection of the abnormality. However, we have confirmed that MRI features traditionally associated with FCD more frequently appear in the epileptogenic zone and tend to accumulate there, when compared to other brain areas. It is therefore the combination of multiple dysplastic features along with being the largest area affected that distinguishes the epileptogenic zone from other dysplastic regions.

### 4.3 The role of mTOR signaling in TSC and FCD

The fact that the EZ of TSC patients specifically harbors features found in patients with FCD not diagnosed with TSC, may reflect their shared genetic background. TSC arises as a result of germline pathogenic variants in TSC1 or TSC2 genes; dysplastic lesions of brain cortex in TSC patients may harbor second‐hit mutations.^20^ Interestingly, hemimegalencephaly may arise as a result of germline and second‐hit somatic mutation in *TSC2*, without any other organ symptoms of TSC.^21^ Somatic variants in genes of mTOR signaling pathway, including TSC1, TSC2, PIK3CA, MTOR, and AKT3, are found in 63% of FCD type II and hemimegalencephaly tissue samples obtained in resective epilepsy surgery;^22^ some patients with FCD also carry germline pathogenic variants in genes of GATOR1 complex, another regulator of mTOR signaling pathway.^23^ In addition, TSC and FCD share some common histopathological and cytological abnormalities, including the presence of giant and dysmorphic neurons, respectively.^24^ These reports provide substantial evidence for the role of aberrant mTOR signaling in both TSC and FCD, justifying the use of an umbrella term “mTORopathies” that encompasses TSC, FCD, and other mTOR‐associated malformations of cortical development.^25^

### 4.4 Implications for clinical practice in epilepsy surgery planning in TSC patients

First, this study highlights that thorough MRI evaluation is needed to identify these most important features that indicate the EZ in TSC patients. Second, our results show which features need to be actively sought for in the process of MRI evaluation of epilepsy surgery candidates with TSC. From the experience of both epilepsy surgery centers involved,^26^, ^27^ we conclude that detailed and reliable MRI evaluation represents a vital step in the pre‐surgical evaluation of TSC patients. Since TSC patients often require long‐term invasive EEG studies, they may be better tailored and in specific cases even omitted, if the EZ is reliably delineated with non‐invasive methods.

### 4.5 Study limitations

To our knowledge, this is the first study that describes a combination of radiological features that characterize the EZ in TSC patients. Although the size of studied cohort is limited, one needs to consider that TSC patients constitute a relatively minor proportion of epilepsy surgery candidates; we therefore included all post‐surgically seizure‐free patients with TSC in order to present as broad as possible spectrum of pediatric epilepsy surgery patients with TSC.

The retrospective design limited data collection and might have introduced bias in terms of patient selection; however, we included all TSC patients from the two centers who had undergone epilepsy surgery with pre‐ and postoperative high‐quality MRI available. Based on the definition of epileptogenic zone (“the area of cortex that is indispensable for the generation of epileptic seizures”)^28^ and the implication that if a patient becomes seizure‐free after surgery, the epileptogenic zone must have been removed,^28^ we only analyzed the resected areas in the group of patients who were and remained seizure‐free at 2‐year follow‐up. In patients who failed to reach seizure freedom, we could not have ruled out whether the resection was incomplete or whether the presumed EZ was incorrectly localized, and therefore, these patients were excluded from the analyses. Despite our best efforts, we cannot completely rule out that in some patients' seizures might relapse over a longer period after surgery, for example in the process of AED withdrawal. However, a follow‐up period of minimum 2 years has traditionally been considered sufficient when reporting seizure outcome in surgical series.^29^ Also, our approach implies that some non‐epileptogenic tissue may have been resected beyond the margins of the “true” epileptogenic zone, even though the resections were tailored to include the minimum amount of brain tissue required to achieve freedom from seizures and spare functional cortex and subcortical tracts. As mentioned previously, structural MRI guides and informs the process of identification of the EZ, but its correct delineation can only be achieved when combining structural and functional neuroimaging, non‐invasive, and invasive neurophysiological and neuropsychological methods.

The variability of MRI protocols used represents another obvious limitation. Indeed, the MRI protocols change over time and so does the technical quality of MRI. Our neuroradiologists agreed on minimal technical requirements for MRI protocols that would enable them to detect distinct dysplastic features. Given that all patients were epilepsy surgery candidates, they all had the most advanced MRI protocols at the time of epilepsy surgery planning. Combined with the lengthy expertise in epilepsy surgery of both centers involved, eventually, all MRI sequences, including those from the earliest study period, met the criteria for reliable MRI evaluation.

## 5 CONCLUSION

The epileptogenic zone in TSC patients harbors multiple MRI features traditionally ascribed to FCD, including increased cortical thickness, transmantle sign, and blurring of the gray/white matter, and to TSC, for example, calcifications; it tends to be the largest area affected with the highest number of dysplastic lesions. No single feature but the combination thereof can reliably identify the EZ. Our findings may therefore contribute to the robustness in the process of pre‐surgical evaluation of MRI in epilepsy surgery candidates with TSC.

## CONFLICT OF INTEREST

None of the authors has any conflict of interest to disclose.
